# New York Odontological Society

**Published:** 1886-06

**Authors:** 


### NEW YORK ODONTOLOGICAL SOCIETY.

---

The regular meeting of this society for the month of May was held on the 11th ult., in the parlors of the Academy of Medicine, the President, Dr. E. A. Bogue, in the chair.

*Dr. J. Morgan Howe*—Read a paper from Dr. C. F. Ives, in which the attention of the society was called to a new remedial agent known as iodol, a substitute for iodoform, and which, the paper claimed, was in many respects superior to iodoform in the treatment of devitalized teeth.

*Dr. H. C. Meriam*, of Salem, Mass. — Exhibited some new forms of artificial crowns, and read a paper describing his method of applying them to the roots. He also exhibited disks formed by a combination of corundum and flux carried to a high temperature. These, he stated, were very efficient in shaping the porcelain crowns to fit the roots, and the doctor gave diagrams on the blackboard to illustrate the manner in which his crowns were adjusted. He also presented a number of drills, the composition of which he was unable to give, but which much resembled the "diamond drill." These were useful for enlarging the canals of artificial crowns, and Dr. Meriam considers them far superior to anything in the market for this purpose. The paper contained some very good points of a practical nature, but was not discussed by the members.

*Dr. Geo. T. Allan*—Followed with the continuation of a previous paper upon "Caries of Teeth," with illustrations on the screen. The paper was discussed by Drs. Hodson, Perry, Bogue, Rich, Dwinelle, Raymond, Littig and Northrop.

The chief points of discussion were as to whether or not decalcification of the dentine would still go on in a well prepared cavity under a perfectly tight filling, from the presence of bacteria. Both the essayist and Dr. Rich were inclined to the opinion that the decay would continue, and cited Prof. Miller's views as evidence. Dr. Dwinelle and others seemed to think that, years ago, it was demonstrated that the decay could not go on in well filled cavities; also that the micro-organisms could be destroyed by the use of carbolic acid, or some other antiseptic agent.

*Dr. Perry*—Could not agree with the essayist in his method of anticipating caries by cutting away the proximal surfaces of teeth. This should be resorted to only in exceptional or rare instances, and then only in the front teeth. He thought the time had passed for the profession to sanction the cutting away of healthy tooth structure, and he believed that decayed teeth should in every instance be restored to their original shapes, as far as possible, by contour fillings.

*Dr. Hodson*—Remarked that some years ago he was induced in several instances to cut away the proximal surfaces of teeth in what he termed an "ideal" manner. His efforts, however, had been rewarded by the presence of *real cavities* in every case. He felt it to be one of the greatest sins he had ever committed during his professional career.

